# Hepatitis B and C infections in HIV-1 and non-HIV infected pregnant women in the Brong-Ahafo Region, Ghana

**DOI:** 10.1371/journal.pone.0219922

**Published:** 2019-07-19

**Authors:** Margaret T. Frempong, Paul Ntiamoah, Max Efui Annani-Akollor, William K. B. A. Owiredu, Otchere Addai-Mensah, Eddie-Williams Owiredu, Denis Adu-Gyasi, Evans Owusu Agyapong, Lorraine Sallah

**Affiliations:** 1 Department of Molecular Medicine, School of Medical Sciences, College of Health Sciences, Kwame Nkrumah University of Science and Technology, Kumasi, Ghana; 2 Department of Medical Diagnostics, Faculty of Allied Health Sciences, College of Health Sciences, Kwame Nkrumah University of Science and Technology, Kumasi, Ghana; 3 Kintampo Health Research Centre, Kintampo, Ghana; 4 St. Elizabeth Hospital, Hwidiem, Brong-Ahafo Region, Ghana; 5 Department of Physiology, School of Medical Sciences, College of Health Sciences, Kwame Nkrumah University of Science and Technology, Kumasi, Ghana; University of Cincinnati College of Medicine, UNITED STATES

## Abstract

**Background:**

Hepatitis B (HBV) or hepatitis C (HCV) virus co-infections in HIV are alarming during pregnancy due to the risk of vertical transmission and the eventual adverse effects on neonates. This study was conducted to ascertain the sero-prevalence of HIV/HBV and HIV/HCV co-infections, evaluate the effect of the co-infections on the immunological and virological characteristics and assess the association between some demographic and lifestyle characteristics and risk of HBV, HCV, HIV/HBV and HIV/HCV co-infections among pregnant women living in the Brong-Ahafo Region of Ghana.

**Methods:**

This comparative cross-sectional study was conducted at the anti-retroviral therapy (ART) clinics of the St. Elizabeth Hospital and the Holy Family Hospital, Brong-Ahafo Region, Ghana. A total of 248 consecutive consenting pregnant Ghanaian women, 148 diagnosed with HIV [HIV (+)] and 100 who were HIV negative [HIV (-)], were recruited. Validated questionnaire was used to obtain demographic and lifestyle data. Venous blood samples were obtained and HCV status, HBV profile, CD4+ T cell count, and HIV-1 RNA load were determined.

**Results:**

The sero-prevalence of HIV (+) /HBV, HIV (+) /HCV, HIV (-)/HBV, and HIV (-)/HCV infections were 22 (14.9%), 6 (4.1%), 10 (10.0%), and 12 (12.0%) respectively. HIV-1 viral load was not significantly different between HIV/HBV, HIV/HCV co-infection and HIV mono-infection. However, CD4+ T lymphocyte count (364 vs 512 vs 514 cells/μl; p = 0.0009) was significantly lower in HIV/HBV co-infection compared to HIV/HCV and HIV mono-infection respectively. There was no significant association between demographic and lifestyle characteristics and risk of HBV and HCV infections in HIV positive and negative subjects except for late diagnosis of HIV and history of sharing razors blades and pins, where increased odds of HIV (+) /HBV and HIV (-)/HBV infection were observed.

**Conclusions:**

The prevalence of HIV (+)/HBV (14.9%), HIV (+)/HCV (4.1%), HIV (-)/HBV (10.0%), and HIV (-)/HCV (12.0%) are high among pregnant women in the Brong Ahafo Region of Ghana. HIV/HBV is associated with reduced CD4+ T lymphocyte count but not HIV-1 viral load. Early diagnosis of HIV and intensification of routine antenatal HBV and HCV are essential to abate the risk of maternal to child transmission.

## Introduction

Human Immunodeficiency virus (HIV), Hepatitis B virus (HBV) and Hepatitis C virus (HCV) are the predominant cause of chronic viral infections globally [1]. The WHO Global Health Observatory reports that approximately 36.9 million people are infected with HIV worldwide, with more than half of these being women and 1.8 million being children [2]. Most of these HIV infected individuals are from low- and middle- income countries, and it is estimated that 66% of them are living in sub-Saharan Africa [3, 4]. Currently in Ghana, 310 000 people are living with HIV, of which 190 000 are women [4]. In the Brong Ahafo region of Ghana, an estimated number of adults living with HIV is 28,577, of which 20,455 are females [5].

HIV infected individuals are predisposed to other infections such as tuberculosis which can make the management of the disease arduous [3]. Reports show that, globally, approximately 325 million people are infected with HBV and HCV, and 1.34 million of these individuals expire annually [6]. The estimated number of individuals infected with HCV is 130–150 million [7] and about 2.3 million HIV infected individuals are co-infected with HCV; thus accounting for 6.2% of people living with HIV globally. In sub-Saharan Africa, the prevalence of HIV/HCV co-infection is estimated at 2.98–4.34% [8]. Additionally, the estimated prevalence of HBV surface antigen is 6.1% worldwide despite the introduction of universal hepatitis B vaccination and potent antiviral therapy [9]. HBV infection is endemic in sub-Saharan Africa and the prevalence of HIV/HBV co-infection in sub-Saharan Africa is between 6% and 25% [10]. In Ghana, the prevalence ranges between 8 and 15% [11], of which 6.4% and 15.6% are pregnant women and children respectively [12]. Co-infection of HBV and HCV in HIV infected persons is attributed to mutual routes of transmission [13].

Individually, HIV, HCV and HBV cause chronic conditions. However, in co-infection states, they may lead to life-threatening and fatal conditions especially during pregnancy where there is a high risk of maternal complications and vertical transmission which is associated with foetal and neonatal hepatitis [14–16]. The clinical management of HIV/HBV and HIV/HCV co-infected individuals is challenging and evidence suggests that coinfection with HBV or HCV adversely affect the prognosis of HIV infection and results in complex interactions with antiretroviral therapy [17]. Despite the effect of HBV and HCV infections among pregnant women (e.g. cholestasis of pregnancy, gestational diabetes etc.) as well as their babies (e.g. preterm birth, low birth weight neonatal abstinence syndrome etc.) [18, 19], limited studies have addressed the issue of co-infection with HCV and/or HBV in HIV-positive pregnant women globally to date, with no study conducted in Ghana. It is against this background that this study was conducted to ascertain the sero-prevalence of HBV, HCV, HIV/HBV and HIV/HCV, to evaluate the effect of the co-infections on the immunological and virological characteristics in pregnant women and to identify the association between some demographic and lifestyle characteristics and risk of HBV and HCV infections in HIV positive and negative pregnant women in the Brong-Ahafo Region of Ghana.

## Materials and methods

### Study design/setting

This was a comparative cross-sectional study conducted at the antiretroviral therapy (ART) clinic of the St. Elizabeth Hospital, Hwidiem and the Holy Family Hospital, Techiman, all in the Brong-Ahafo Region of Ghana between May 2012 and 2013. Both hospitals are located in a semi-urban setting. The St. Elizabeth hospital is a Catholic health delivery facility which serves as the main district and referral hospital for both Asutifi North and South districts, offering antiretroviral therapy (ART), voluntary counseling and testing/ Prevention of Mother to Child Transmission (VCT/PMTCT) services. These services are provided in accordance with the WHO recommendations formulated for the 2013 guidelines on HIV testing and counselling, antiretroviral therapy (ART) and HIV service delivery [20]. The hospital also offers other healthcare services including curative, preventive, rehabilitative, diagnostic, and special programs to all its neighboring communities. The Holy Family Hospital is a 140 bed Missions hospital that serves the people of Techiman and the neighboring towns. The clients served by the Holy Family Hospital are predominantly farmers and traders.

### Participants’ recruitment

**Fig 1:**
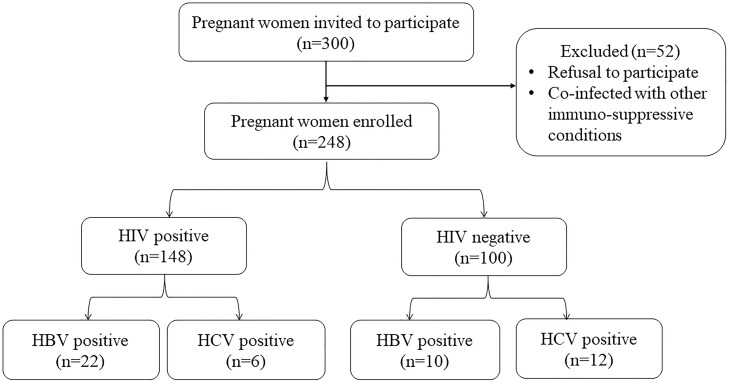
Flowchart of the protocol for the selection of subject.

The sample size for the study was calculated using Fischer’s sampling formula (*N* = *Z*^2^ *PQ*/*d*^2^), where *Z* is the critical value of the normal distribution (1.96 at 95% CI); *P* is the estimated prevalence of HIV among pregnant women in Ghana (2.4%) [21]; *d* is the absolute precision or sampling error tolerated = 5%. From the above equation, the minimum sample size was 36 participants for each group. However, in the effort to enhance the statistical power, a total of 248 consecutive consenting pregnant Ghanaian women comprising 148 diagnosed with HIV and 100 who were HIV negative, living in the Brong-Ahafo Region were recruited during their routine clinic visit days. All HIV patients enrolled were on antiretroviral therapy (ART) at the time of study. Socio-demographic data were collected using an investigator-administered validated questionnaire in a language that they could easily comprehend. Additional information relevant to the study objectives were retrieved from the hospitals archive. Participants’ selection protocol is shown in Fig 1.

### Inclusion/ Exclusion criteria

Only pregnant women were included in this study. HIV positive pregnant women who have co-morbidities such as cancer, tuberculosis, and other immuno-suppressive conditions were excluded from this study.

### Sample collection and assay

Venous blood sample (5 ml) was obtained from each participant under aseptic conditions. Haemoglobin level, total white cell and platelet count was performed using the Sysmex KX N21 analyzer (Symex Corporation, Japan). Screening for HIV was performed using the First Response HIV-1/HIV-2 WB (PMC Medical India Pvt Ltd) and confirmed with OraQuick ADVANCE Rapid HIV-1/2 (OraSure Technologies, Inc.). In the case of discordant results, Enzyme-Linked Immunosorbent Assay (ELISA; Organon Teknika, Boxtel, Co., Ltd., Netherlands) was used to break the tie. All HIV positives included in this study were HIV-1. CD4+ T cell count and HIV-1 RNA loads were estimated using BD FacsCalibur analyzer (BD Biosciences, USA) and COBAS AmpliPrep/COBAS TaqMan Analyzer (lower limit of detection: <20 copies/ml) (Roche Diagnostics, USA) respectively. HBV profile and anti-HCV antibody testing were done using commercially available test kits (Guangzhou Wondfo Biotech Co. Ltd, Luogang District, China). The HBV profile kit determined HBV surface antigen (HBsAg), HBV surface antibody (HBsAb), HBV ‘e’ antigen (HBeAg), HBV ‘e’ antibody (HBeAb), and HBV core antibody (HBcAb) based on Immunochromatographic technique. The testing, reporting and interpretation of the results were performed following the manufacturer’s instructions. To ensure accuracy, two each of the HBV profile and anti-HCV kits were pre-tested with known positive and negative samples prior to testing of participants samples. Samples positive for HBV using the HBV profile kits were further confirmed for the presence HBsAg using the DRG ELISA kit (DRG International, Inc, New Jersey, USA) following the manufacturer’s specifications. Samples confirmed reactive by the DRG ELISA kit were considered positive for HBsAg.

### Data analysis

All categorical data were presented as frequencies (percentages) and Chi square and Fishers exact test statistic were used to test for associations between the baseline characteristics and HIV infection where applicable. Continuous variables were presented as mean ± SD and significance of difference between groups were tested with independent sample t-test. One-way ANOVA, followed by Tukey post-hoc multiple comparison test was used to assess the significance of differences of HIV-1 viral load and CD4 count between HIV/HBV, HIV/HCV, and HIV mono-infection. Univariate logistic regression analysis was performed to determine the odds of the presence of serological markers in HIV-positive compared to the HIV negative participants. Multivariate logistic regression analysis, using the enter method for variables with *p* values < 0.25 after univariate analysis, was performed to identify possible risk factors for HBV and HCV infections in HIV positive and HIV negative pregnant women. All tests were two-sided and a *p* value < 0.05 was considered statistically significant. All statistical analyses were performed using IBM Statistical Package for the Social Sciences (SPSS) software 25 (SPSS Inc., Chicago, IL, USA), and GraphPad Prism 7 version 7.04 (GraphPad Software, Inc., La Jolla, California USA).

### Ethics

Ethical approval for this study was obtained from the Committee on Human Research Publication and Ethics (CHRPE) of the School of Medical Sciences, Kwame Nkrumah University of Science and Technology and from the Research (CHRPE/RC/131/12) and Development Units of St. Elizabeth Hospital, Hwidiem and Holy Family hospital, Techiman. Written informed consent was obtained from all participants who opted to participate after the aims and objectives of the study was explained to them. Participation was voluntary, and respondents were assured that the information obtained was strictly for research and academic purposes only and were guaranteed the liberty to opt out from the study at their own convenience.

## Results

Of the 248 participants recruited, 148 (59.7%) were HIV positive with mean age of 29.1 years whereas 100 (40.3%) were HIV negative with a mean age of 28.9 years. Overweight, reduced haemoglobin, and low WBC count were significantly associated with subjects with HIV compared to those without HIV. A higher proportion of the HIV-infected participants had history of sharing razors blades and pins compared to subjects without HIV (Table 1).

**Table 1:** Baseline characteristics of study population.

Variables	HIV-Positive n = 148 (59.7%)	HIV-Negative n = 100 (40.3%)	P-value
**Age (years)**	29.1 ± 6.1	28.9 ± 6.4	0.804*
15–19	14 (46.7)	16 (53.3)	0.299†
20–30	82 (61.2)	52 (38.8)	
>30Years	52 (61.9)	32 (38.1)	
**History of sharing razors blades and pins**			**<0.0001**‡
Ever	60 (78.9)	16 (21.1)	
Never	88 (51.1)	84 (48.9)	
**History of blood transfusion**			0.408‡
Ever	28 (77.8)	8 (22.2)	
Never	120 (56.6)	92 (43.4)	
**Period of HIV diagnosis**			N/A
Before pregnancy	34 (23.0)	-	
During pregnancy	114 (77.0)	-	
**If during pregnancy, at what stage?**			N/A
First trimester	14 (12.3)	-	
Second trimester	60 (52.6)	-	
Third trimester	40 (35.1)	-	
**Medication used**			
3TC+AZT+EFV	4 (2.7)	-	N/A
3TC+TDF+EFV	12 (8.1)	-	N/A
3TC+AZT+NVP	132 (89.2)	-	N/A
**Marital status**			**0.039‡**
Single	44 (71.0)	18 (29.0)	
Married	104 (55.9)	82 (44.1)	
**Employment status**			**0.007**†
Formal	14 (50.0)	14 (50.0)	
Informal	100 (56.2)	78 (43.8)	
Unemployed	34 (81.0)	8 (19.0)	
**BMI (kg/m**^**2**^**)**	26.90 ± 3.91	23.17 ± 3.55	<0.0001*
Underweight	16 (72.7)	6 (27.3)	**<0.0001**†
Normal	64 (45.7)	76 (54.3)	
Overweight	68 (79.1)	18 (20.9)	
**Haematology**			
Haemoglobin (g/dl)	9.7 ± 1.81	10.75 ± 1.68	<0.0001*
WBC (10^3^/μL)	5.60 ± 1.81	7.23 ± 2.13	<0.0001*
Platelet count (10^3^/μL)	220.62 ± 86.73	209.38 ± 65.59	0.272*

Continuous data is presented as Mean ± SD. Categorical data is presented as frequency (%).

Chi square† and Fisher‡ exact test was performed to compare categorical variables.

*Independent t-test was performed to compare continuous variables.

Lamivudine (3TC), Zidovudine (AZT), Nevirapine (NVP), Tenofovir (TDF), Efavirenz (EFV). N/A: Not applicable, *p* < 0.05 was considered statistically significant (*p* values of significant variables are in bold print).

**Fig 2:**
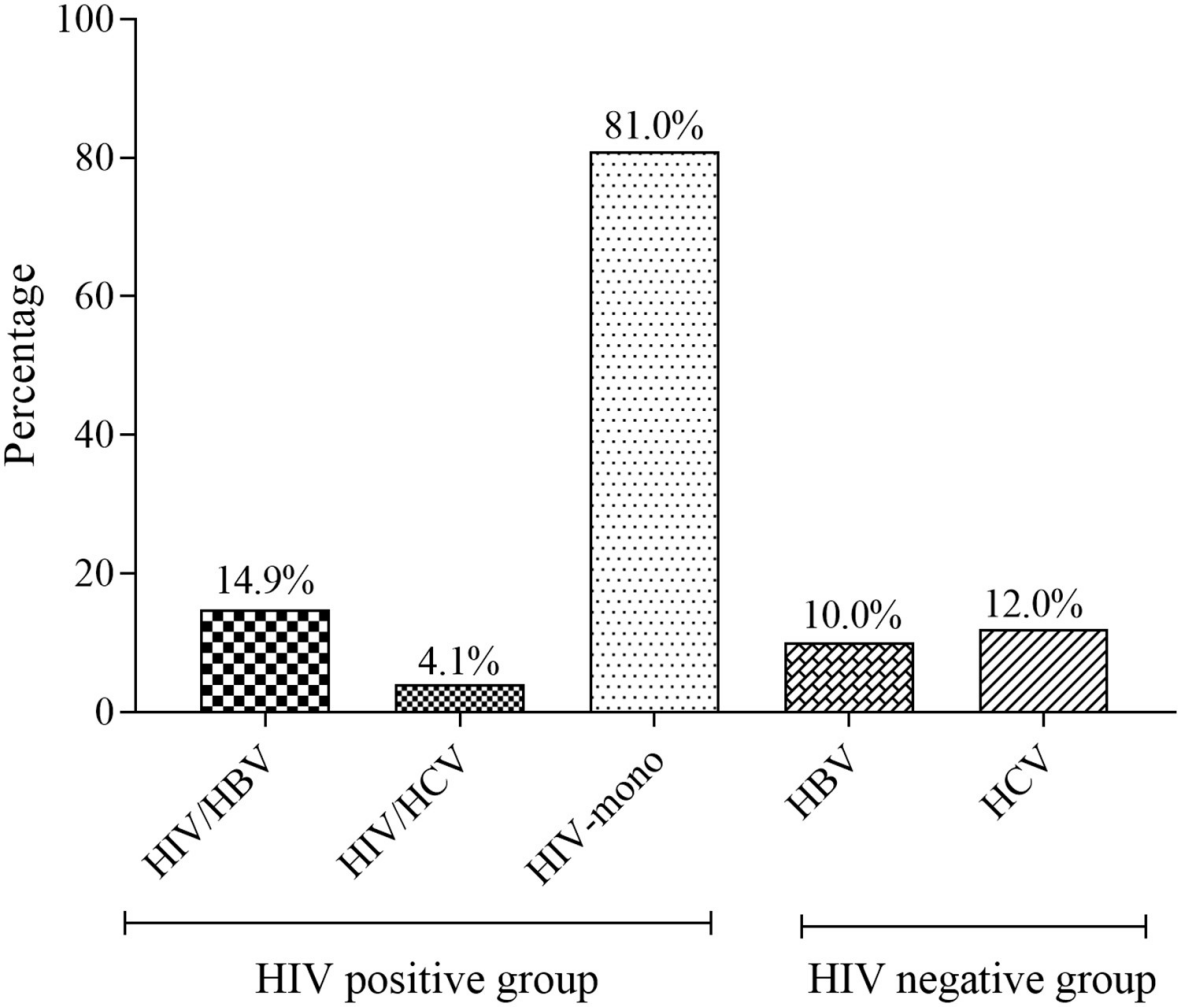
The sero-prevalence of HBV and HCV among the study population.

The sero-prevalence of HIV/HBV and HIV/HCV was 14.9% and 4.1% respectively. Among the HIV-negative pregnant women, the prevalence of HBV and HCV was 10.0% and 12.0% respectively. There was no triple infection (Fig 2).

Of the 22 HIV positive patients who were positive for HBsAg, 4 (18.2%) were positive for HBeAg. Additionally, HIV-infected participants were significantly less likely to sero-convert from HBeAg to HBeAb [OR = 0.21, 95% CI (0.10–0.43), p<0.0001]. On the other hand, HIV-positive participants were significantly less likely to be infected with HCV [OR = 0.31, 95% CI (0.11–0.86), p = 0.024] (Table 2).

**Table 2:** The prevalence of HBV and HCV serological markers stratified by the HIV status.

Serological Marker	HIV-Positive n = 148 (59.7%)	HIV-Negative n = 100(40.3%)	OR (95% CI)	P-value
HBsAg	22 (68.8)	10 (31.3)	1.57 (0.71–3.48)	0.265
HBsAb (Anti-HBs)	4 (50.0)	4 (50.0)	0.67 (0.16–2.73)	0.573
HBeAg	4 (100.0)	0 (0.0)	6.26 (0.33–117.56)	0.220
HBeAb (Anti-Hbe)	12 (28.6)	30 (71.4)	0.21 (0.10–0.43)	**<0.0001**
HBcAb (Anti-HBc)	44 (57.9)	32 (42.1)	0.90 (0.52–1.56)	0.704
Anti-HCV	6 (33.3)	12 (66.7)	0.31 (0.11–0.86)	**0.024**

Univariate logistic regression analysis was performed to determine the odds of the presence of serological markers in HIV-positive compared to the HIV negative participants. *p* < 0.05 was considered statistically significant (*p* values of significant variables are in bold print).

HIV-1 viral load was not significantly different between HIV/HBV, HIV/HCV co-infection and HIV mono-infection. However, CD4+ T lymphocyte count was significantly lower in HIV/HBV co-infection compared to HIV/HCV and HIV mono-infection respectively (Table 3).

**Table 3:** Virological and cellular markers of HIV among HIV positive participants.

Parameters	HIV/HBV (a)	HIV/HCV (b)	HIV-mono (c)	p-value	Significant pairs
HIV-1 viral Load (log_10_ copies/ml)	4.53 ± 3.70	2.72 ± 2.11	4.45 ± 3.72	0.523	-
CD4 Count (cells/μl)	364 ± 181	512 ± 123	514 ± 169	**0.0009**	a&b; a&c

One-way ANOVA followed by Tukey post hoc multiple comparison test was used to test for significance of difference between HIV/HBV, HIV/HCV co-infections and HIV mono-infection. *p* < 0.05 was considered statistically significant (*p* values of significant variables are in bold print).

Multivariate logistic regression analysis showed no statistically significant association between age, marital status, employment, history of sharing razors blades and pins, and history of blood transfusion with risk of HIV/HBV and HIV/HCV co-infection. However, late diagnosis of HIV (during pregnancy) was significantly associated with increased odds of HIV/HBV [aOR = 3.02 (0.16–14.92–306.5), p = 0.015] compared to HIV diagnosis before pregnancy. Similarly, there was no significant association between the risk factors and HBV and HCV infection in HIV negative pregnant women with the exception of having history of sharing razors blades and pins, where a significantly increased odd of HBV infection [aOR = 6.47, 95% CI (1.15–36.48), p = 0.030] was observed (Table 4).

**Table 4:** Multivariate logistic regression analysis of risk factors for HBV and HCV co-infections in HIV positive and HIV negative pregnant women.

Risk Factors	HIV positive				HIV negative			
	HBV		HCV		HBV		HCV	
	aOR (95% CI)	p-value	aOR (95% CI)	p-value	aOR (95% CI)	p-value	aOR (95% CI)	p-value
**Age group (years)**								
15–19	1		1		1		1	
20–30	1.20 (0.24–6.58)	0.794	0.81 (0.04–18.63)	0.900	1.02 (0.08–8.93)	0.870	3.85 (0.25–60.52)	0.338
>30	0.52 (0.07–3.85)	0.525	23.16 (0.31–178.94)	0.150	1.11 (0.12–10.34)	0.930	11.23 (0.82–153.0)	0.070
**Marital status**								
Single	1		1		1		1	
Married	0.49 (0.18–1.35)	0.169	0.04 (0.02–0.45)	0.310	0.35 (0.04–2.83)	0.330	0.32 (0.06–1.66)	0.173
**Employment status**								
Unemployed	1		1		1		1	
Employed	0.98 (0.30–3.16)	0.971	0.13 (0.0–0.53)	0.220	0.57 (0.05–6.73)	0.650	0.44 (0.05–3.76)	0.454
**History of sharing razors blades and pins**								
Never	1		1		1		1	
Ever	2.38 (0.83–6.78)	0.105	9.56 (0.68–134.55)	0.090	6.47 (1.15–36.48)	**0.030**	3.57 (0.62–20.53)	0.154
**History of blood transfusion**								
Never	1		1		1		1	
Ever	1.77 (0.50–6.36)	0.379	5.98 (0.43–84.30)	0.190	4.62 (0.55–39.19)	0.160	1.09 (0.12–9.95)	0.942
**Period of HIV diagnosis**					-	-	-	-
Before pregnancy	1		1					
During pregnancy	3.02 (0.61–14.92)	**0.015**	0.04 (0.0–0.45)	0.900				
**If during pregnancy, at what stage?**					-	-	-	-
First trimester	1		1					
Second trimester	7.47 (0.42–134.1)	0.172	0.24 (0.01–12.59)	0.480				
Third trimester	10.06 (0.54–187.5)	0.122	2.38 (0.11–52.77)	0.580				

Multivariate logistic regression analysis was performed to determine the odds of the sociodemographic, lifestyle and obstetric factors HBV and HCV infections in HIV positive and negative pregnant women. *p* < 0.05 was considered statistically significant (*p* values of significant variables are in bold print).

## Discussion

HBV or HCV co-infection is a major health burden among HIV patients even in the era of Highly Active Antiretroviral therapy (HAART). These co-infections are even more alarming during pregnancy due to the risk of vertical transmission and the eventual adverse effects on the newborns [18, 19]. In Ghana however, no current study has addressed the issue of co-infection with HCV and/or HBV in HIV-infected pregnant women to date. Thus, the need for this study to provide update on this issue.

We found a high sero-prevalence of HBV (14.9%) and HCV (4.1%) infection among HIV positive pregnant women. This finding is similar to the finding of a study by Simpore *et al*. [15] among pregnant women in Burkina Faso. They reported a sero-prevalence of HIV/HBV and HIV/HCV of 11.6% and 4.8% respectively. On the contrary, our finding is higher than the findings of Rouet *et al*. [22] who reported sero-prevalence of HBV and HCV of 9.0% and 1.0% respectively among HIV-positive pregnant women in Côte d’Ivoire. This discrepancy could be due to the higher national HBV and HCV prevalence rates for women in Ghana (HBV: 12.3% and HCV: 3.2%) [23, 24] compared to Côte d’Ivoire (HBV: 9.9% and HCV: 2.2%) [24, 25]. Sagoe *et al*. [26] also reported the sero-prevalence of 13.0% and 3.6% for HBV and HCV respectively people living with HIV in Ghana; a finding similar to the findings of this present study. Additionally, the prevalence of HBV (10.0%) and HCV (12.0%) among HIV negative pregnant women in this study is similar to the finding of a study by Rouet *et al*. [22] among Ivorian women and Kumar *et al*. [27] among Egyptian women respectively. They reported HBV and HCV prevalence of 8.0% and 13% respectively in HIV negative women. Nonetheless, the higher sero-prevalence of HCV among HIV negative pregnant women compared to HBV is in dissonance with some findings of studies conducted locally [28, 29], and internationally [30]. This discrepancy may be due to fact the aforementioned studies utilized ELISA-based methods for the detection of HCV antibodies, which have higher specificity compared to the Immunochromatographic method employed in this study. Furthermore, the higher sero-prevalence of HIV/HBV compared to HIV/HCV in this study is also consistent with studies by Simpore *et al*. [15], Rouet *et al*. [22], and Sagoe *et al*. [26]. However, a study by Landes *et al*. [31] in Europe reported a prevalence of 4.9% and 12.3% for HBV and HCV co-infections among HIV-infected pregnant women respectively, showing a higher prevalence of HIV/HCV than HIV/HBV. The discrepancy with our finding may be due to differences in geographical location and lifestyle. Moreover, Europe has been reported to have a higher prevalence of HCV than HBV [32] compared to sub-Saharan Africa where the prevalence of HBV is usually higher than HCV.

Taken together, the sero-prevalence of HBV was higher among pregnant women with HIV (14.9%) than pregnant women without HIV (10.0%); a finding which is consistent with the finding of a study by Simpore *et al*. [15] in Burkina Faso and Rouet *et al*. [22] in Côte d’Ivoire. The high prevalence of HBV in HIV is attributed to common routes of transmission [33, 34]. Furthermore, the high prevalence of HBV among pregnant women also suggests these pregnant women may serve as a possible potential pool of HBV to fuel hepatitis B endemicity in Ghana. Nonetheless, the low prevalence of HBeAg among HIV/HBV co-infected pregnant women and the fact that none of the HIV negative-HBV positive pregnant women in this study were positive for HBeAg suggest a low likelihood of perinatal transmission of HBV in Ghana, because perinatal transmission of HBV is largely effective in HBeAg positive mothers [35].

While some studies have reported a higher HIV viral load among HIV/HBV co-infected subjects’ relative to HIV/HCV and HIV mono-infection [33, 36], others report no significant association [37–39]. In this study, we observed no significant differences regarding HIV-1 viral load between pregnant women with HIV/HBV, HIV/HCV, and HIV mono-infection. However, it worthy of note that, despite being on ART, the viral loads of all the HIV positive subjects were high. This could possibly be linked to the high prevalence of non-adherence to ART among HIV patients in Ghana [40–42]. On the contrary, pregnant women with HIV/HBV presented with significantly lower CD4+ T-lymphocyte in comparison with HIV/HCV and HIV mono-infection respectively, which is in harmony with the finding of a study by Laurent *et al*. [38] and Omland *et al*. [39].

In assessing the risk factors for HIV/HBV and HIV/HCV, we found no significant association between age, marital status, employment, history of sharing razors blades and pins, and history of blood transfusion with risk of HBV and HCV infection among HIV positive pregnant women as consistent with a cross-sectional study by Zenebe *et al*. [43] in Ethiopia and Nimzing *et al*. [44] in Nigeria. However, late diagnosis of HIV (during pregnancy) posed greater risk of acquiring HIV/HBV compared to HIV diagnosis before pregnancy. This underscores the need for the development and implementation of a comprehensive policy for the screening of all females of reproductive age in the study area. Furthermore, there is the need to ensure early diagnosis of HIV during pregnancy since the risk of co-infection with HBV was also high when HIV was diagnosed in the second and third trimesters compared to the first trimester. On other hand, among HIV negative subjects, we observed that having history of sharing razors blades and pins, compared to those with no history, posed a significantly higher risk of HBV infection. This was expected because sharing sharps has been established as a risk factor for HBV infection [45]. This finding is partly in harmony with the finding of Ngaira *et al*. [46] among pregnant women attending antenatal clinic in Kenya and Obi *et al*. [47] among pregnant women in Nigeria.

Despite the non-significant associations, it is worth noting that, pregnant women aged 20–30 years old, those who have history of sharing razors blades and pins and blood transfusion had higher odds of HIV/HBV, HIV/HCV co-infections and HBV, HCV infections in HIV negative pregnant women. The higher risk of co-infection among women aged 20–30 years old could be attributed to the increased sexual activities among the youth. Furthermore, sharing razors blades and pins and blood transfusion has been reported to increase the risk of infections such as HIV, HBV and HCV. As such, public health education on safe sex, especially for young women, needs to be strengthened, and haemovigilance must be intensified to reduce and possibly eradicate transfusion-transmitted infections, particularly during pregnancy where there is increased demand for blood.

This study is nonetheless limited the fact that it was conducted in a semi-urban setting and thus the findings may not be generalizable to other areas especially rural areas. Furthermore, only a single brand of RDT was used to evaluate the prevalence of HCV and for the HBV profile. As such, the prevalence may not be the same when other commercially available test kits are employed. In addition, we did not perform anti-HBc IgM. Also, testing for HCV based on detection of antibodies rather than HCV RNA and the fact that we did not perform HBV DNA testing may have over-estimated the prevalence of HCV and HBV in this study. Additionally, the lower number of co-infected subjects obtained in this study limits some of the formal analysis. Thus, we recommend that further studies be conducted in the larger population.

## Conclusion

The prevalence of HIV/HBV (14.9%), HIV/HCV (4.1%) co-infection, HBV (10.0%), and HCV (12.0%) are high among pregnant women in the Brong Ahafo Region of Ghana. HIV/HBV is associated with reduced CD4+ T lymphocyte count. Early diagnosis of HIV and intensification of routine antenatal HBV are essential to abate the risk of maternal to child transmission.

## Supporting information

S1 Dataset Excel sheet of dataset on which the conclusions of this manuscript were made. (XLSX)
